# Barriers and Facilitators in the Implementation of the Systematic Medical Appraisal, Referral, and Treatment (SMART) Mental Health Digital Intervention in Rural India: Mixed Methods Process Evaluation Study

**DOI:** 10.2196/89164

**Published:** 2026-05-07

**Authors:** Ankita Mukherjee, Mercian Daniel, Sudha Kallakuri, Siddhardha Devarapalli, Sandhya Kanaka Yatirajula, Amanpreet Kaur, Praveen Devarsetty, Usha Raman, Beverley M Essue, Rajesh Sagar, Shashi Kant, Shekhar Saxena, Graham Thornicroft, Anushka Patel, David Peiris, Pallab K Maulik

**Affiliations:** 1 George Institute for Global Health New Delhi, Delhi India; 2 George Institute for Global Health Hyderabad, Telangana India; 3 Jindal School of Psychology & Counselling OP Jindal Global University Sonipat, Haryana India; 4 Faculty of Medicine and Health University of New South Wales, Sydney Sydney, New South Wales Australia; 5 Prasanna School of Public Health Manipal Academy of Higher Education Manipal, Karnataka India; 6 Department of Communication University of Hyderabad Hyderabad, Telangana India; 7 Institute of Health Policy, Management and Evaluation University of Toronto Toronto, ON Canada; 8 Department of Psychiatry All India Institute of Medical Sciences New Delhi, Delhi India; 9 Department of Community Medicine All India Institute of Medical Sciences New Delhi, Delhi India; 10 Harvard T H Chan School of Public Health Harvard University Boston, MA United States; 11 Institute of Psychiatry, Psychology and Neuroscience King's College London London, England United Kingdom; 12 The George Institute for Global Health Sydney, New South Wales Australia

**Keywords:** anxiety, electronic decision support system, LMIC, low- middle-income country, India, mental health depression, mHealth, process evaluation, stigma

## Abstract

**Background:**

An estimated 150 million people have mental health care needs in India, but only 15% are able to access care. Depression and anxiety contribute to a large proportion of mental morbidity. The Systematic Medical Appraisal, Referral, and Treatment (SMART) Mental Health trial used a mobile-based clinical decision support system for primary care doctors and community health workers (CHWs) to identify and treat people at risk of depression, anxiety disorders, and self-harm. A community-based antistigma campaign was also delivered. The intervention led to improved remission rates for depression and anxiety and lower stigma scores.

**Objective:**

A process evaluation assessed (1) implementation fidelity, barriers, and facilitators; (2) perceptions of doctors and CHWs on the use of SMART Mental Health; and (3) the causal pathways that led to trial outcomes.

**Methods:**

A mixed methods evaluation combining backend program data and qualitative data was conducted. A total of 38 focus group discussions and 37 key informant interviews were conducted with primary doctors, CHWs, government officials, local community leaders, and research project staff. The data were coded and analyzed using a framework analysis approach based on the UK Medical Research Council guidance on process evaluations and the Reach, Effectiveness, Adoption, Implementation, and Maintenance framework.

**Results:**

The intervention had high implementation fidelity. Across clusters, the median proportion of participants with at least 1 CHW follow-up was 98% (IQR 96.6%-100%). The referral rate for a psychiatrist was low (224/1697, 13.2%), and only 23.6% (53/224) of those referred visited the psychiatrist. The median exposure to antistigma audiovisual content was 84% (IQR 65.7%-95.9%). At the community level, key implementation barriers included cultural inhibitions in seeking mental health care and the unavailability of patients due to competing demands. Proximity and tight social connections between CHWs and their communities were important facilitators in seeking medical help. Doctor and CHW training, mentoring, and feedback provided by program staff were important facilitators to support the use of the digital health components by the health workforce.

**Conclusions:**

A complex intervention that included both community-based antistigma and clinical digital health interventions achieved high implementation fidelity. Key areas to consider for maintenance of such interventions include (1) the need for sustained community-based strategies to address stigma and other cultural barriers; (2) health workforce strengthening policies, including supportive supervision for CHWs and doctors to increase capability in the use of mental health digital health tools; and (3) strategies to improve access to specialist care for those with more complex care needs.

**Trial Registration:**

Clinical Trial Registry India CTRI/2018/08/015355; https://tinyurl.com/5r63suxp

## Introduction

India has a relatively large burden of mental disorders. A national survey in 2015-2016 found 13.7% lifetime prevalence and 10.6% point prevalence for mental disorders, with an estimated 150 million people in need of mental health care [1]. The Global Burden of Disease Study for India estimates a larger burden, with about 197.3 million people with mental disorders in 2017 [2]. The contribution of common mental disorders (CMDs) to overall disease burden has doubled since 1990 [2]. Despite the very substantial disease burden, the treatment gap for CMDs is 85% [1]. Several factors play a role in this degree of undertreatment. Limited knowledge and stigma related to mental health are major barriers to seeking help [3,4]. There exist substantial gaps in the availability of mental health services at the primary care level, including a paucity of trained mental health professionals, erratic supply of psychotropic medicines in health facilities, inadequate availability of funding for mental health care, and poor planning and use of available funds [5].

The Systematic Medical Appraisal, Referral, and Treatment (SMART) Mental Health study used a mobile health (mHealth) strategy, using a clinical decision support system to enhance the capacity of primary care physicians and village-level community health workers (CHWs) to screen, diagnose, and manage people with CMDs in rural areas. In parallel, a community-based campaign for mental health stigma reduction was used to improve help-seeking for CMDs [6]. The SMART Mental Health cluster randomized controlled trial demonstrated that the intervention significantly reduced depression scores, increased remission of depression and anxiety, and lowered the risk of self-harm. The stigma reduction campaign led to improved knowledge and attitudes related to mental health, and a reduction in overall stigma scores. However, the campaign was not effective in leading to a change in stigma-related behavior scores at the end of the 12-month intervention [7].

While the trial demonstrated beneficial effects on key mental health outcomes, it did not fully explain how and why these effects were achieved, nor why improvements were observed in some domains but not others. In particular, questions remained regarding why gains in stigma-related knowledge and attitudes did not translate into changes in intended behavior toward people with mental illness, what factors facilitated or hindered the integration of digital decision support tools into routine primary care practice by physicians and accredited social health activists (ASHAs), and why service use and referral uptake varied across implementation sites.

Process evaluations are critical for understanding the implementation of complex interventions in real-world settings. They help to understand how interventions are delivered, how contextual factors influence implementation, and how implementation processes shape observed outcomes. This process evaluation was conducted to assess implementation fidelity and better understand barriers and facilitators in implementing the intervention, including potential challenges in scaling up. It further aimed to explore perceptions of doctors and CHWs on the use, effectiveness, and adoption of SMART Mental Health, and the perception of patients about barriers and facilitators in seeking mental health care support.

Conducted prospectively and blinded to trial outcomes, the process evaluation was designed to generate explanatory insight into implementation processes and contextual influences. While it is beyond the scope of this paper to provide causal explanations for all observed trial outcomes, including cluster-level variations or to make definitive causal inference on key active ingredients that led to change, it can provide important insights to interpret the trial results.

## Methods

### The Intervention

The SMART Mental Health intervention combined an electronic decision support system (EDSS) for primary care doctors and CHWs, and a community-based antistigma campaign to manage depression, anxiety, and self-harm risks among adults in rural India. It was implemented across 44 primary health center (PHC) clusters in 2 states—Haryana and Andhra Pradesh—with 22 clusters randomized to the intervention arm and 22 to the control arm.

The intervention had 2 components. The first was an mHealth component. An EDSS was developed for PHC doctors and village-based CHWs known as ASHAs to identify and manage people at high risk of CMDs, referred to in this paper as “high-risk” people.

Following randomization of PHC clusters, individuals identified as “high-risk” in the intervention clusters were regularly followed up by ASHAs and encouraged to seek medical advice. PHC doctors used tablet‑based decision support apps adapted from the World Health Organization’s Mental Health Gap Action Programme Intervention Guide [8] to support clinical decision‑making. The SMART Mental Health platform maintained a registry of high-risk patients, accessible to both ASHAs and doctors within their respective catchment areas. A traffic light–based feature in the app alerted ASHAs about patients who had not visited the doctor or needed additional follow-ups. ASHAs received automated prompts and reminders to support follow‑up on their phones through an interactive voice recording system. PHC doctors attended to patients either at the PHC or at health camps organized in the villages. Those requiring specialist care were referred to psychiatrists at government facilities.

The second component of the intervention was a community-based campaign that used multimedia information, education, and communication (IEC) strategies to enhance knowledge of mental health and reduce stigma, negative attitudes, and improve behaviors toward people needing mental health support. The campaign printed materials, video narratives of people with lived experience, animated and promotional videos featuring local influencers, and live or recorded street‑theater performances in local languages. A detailed description of the intervention has been published elsewhere [6].

The control arm/usual care arm clusters received information on CMDs through pamphlets. Those identified as high-risk were advised to seek care from the PHC doctor or psychiatrist by the ASHA. The antistigma and the mHealth components were not used in the control/usual care arm.

### Conceptual Framework

A detailed protocol for the process evaluation is available [9]. The UK Medical Research Council (MRC) guidance on process evaluation [10] and the Reach, Effectiveness, Adoption, Implementation, and Maintenance (RE-AIM) framework [11] were used to develop key areas of enquiry. The MRC guidance [10] suggests that process evaluations should answer questions related to 3 components: “Context” (how does context affect implementation and outcomes?), “Implementation” (what is delivered and how?), and “Mechanisms of impact” (how does the delivered intervention produce change?). Thus, these 3 components (implementation, mechanism of impact, and context) formed the broad areas of inquiry during the process evaluation. The RE-AIM framework [11] makes use of 5 key areas to evaluate intervention: reach, effectiveness, adoption, implementation, and maintenance. RE-AIM was used to evaluate the “implementation” component of the program. A brief snapshot of how theory was used to guide our line of enquiry is presented in Table 1.

**Table 1:** Conceptual framework for the process evaluation.

Area of enquiry and domains of inquiry			Some key questions
**Context**			
	Differences in context	What are the differences in social, economic, cultural, and health systems between the states and among the clusters? Do contextual differences influence how the program is delivered in different settings?	
	Significant changes in context and program adaptations	What are some of the key contextual factors which influenced the overall implementation of the intervention (eg, COVID-19 pandemic)? What were some of the context-specific adaptations that were made to address emerging challenges?	
	Barriers and facilitators	What are some major barriers faced in implementing the intervention components? What are some of the factors which acted as facilitators in implementation of the intervention components (antistigma campaign, mHealth^a^, training, and capacity building)?	
**Implementation**			
	Implementation fidelity	Was the intervention delivered as planned?	
	Intervention reach	What was the coverage of the different antistigma campaign methods? What was the reach of the mHealth services? Did the ASHAs^b^ face any challenge in reaching out to any category of high-risk individual in their village?	
	Intervention effectiveness	What was the perception of the community and key stakeholders about the use and effectiveness of the antistigma IEC^c^ materials? What was the perception of ASHAs about effectiveness of mHealth in mental health service delivery to manage CMDs^d^ in the community? What is the perception of PHC^e^ doctors about effectiveness of mHealth in mental health service delivery to manage CMDs in the community?	
	Intervention acceptability and adoption	What was the perception of ASHAs about using EDSS^f^ for providing care (challenges, perceived benefits, and potential for routine use of mHealth)? What was the perception of PHC doctors about using EDSS for providing care (challenges, perceived benefits, and potential for routine use of mHealth)?	
	Maintenance (informed by results from posttrial phase)	What was the proportion of ASHAs who continued to provide routine care compared to those who discontinued? What are the cluster-level differences in number of CMD patients provided treatment during the posttrial phase? What are factors that explain differences in adoption or routinization of EDSS in different PHC clusters?	
	Health service use	What are the barriers or facilitators that patients from intervention cluster faced while accessing care in the PHC? How many high-risk individuals identified in the intervention arm did not seek care? What factors can explain this?	
**Mechanism of impact**			
	Variation in outcomes	What are plausible explanations for cluster-level variations observed in the outcomes? What works, for whom, and in what context?	
	Unexpected outcomes	What are some unexpected outcomes, and what factors can be attributed to them?	

^a^mHealth: mobile health.

^b^ASHA: accredited social health activist.

^c^IEC: information, education, and communication.

^d^CMD: common mental disorder.

^e^PHC: primary health center.

^f^EDSS: electronic decision support system.

### Evaluation Methodology

The process evaluation used a mix of quantitative and qualitative data. The quantitative data consisted of program data recorded by the implementation team and stored at the backend of the SMART Mental Health server. These included some key metrics, including follow-up visits made by the ASHAs, doctor consultations made by the study participants, and exposure to various antistigma content.

The qualitative component consisted of focus group discussions (FGDs) and interviews with key stakeholders. Qualitative data were collected in 8 purposively selected clusters out of the 44 clusters. These included 6 intervention clusters and 2 control clusters, equally distributed across the 2 regions. Since the study aimed to focus in-depth on each cluster and a large number of interviews and FGDs were planned for each selected cluster, 8 clusters were deemed feasible and enough to provide rich contextual insights. After discussion with the implementation team, PHC clusters were purposively selected using the principle of maximum divergence, or selecting cases with the most variation to capture a broad spectrum of perspectives and possible reasons behind variations. The following criteria were used for selecting the clusters: (1) clusters where challenges were faced during implementation, (2) clusters where a large proportion of high-risk patients had sought care, and (3) the staff had faced relatively limited challenges in implementation. The purposive overrepresentation of intervention clusters was done to enable a deeper understanding of the implementation of the intervention. This approach necessarily excludes experiences from many clusters and limits comparative inference between intervention and control sites.

Different categories of informants (PHC doctors, ASHAs, high-risk and non–high-risk study cohort, and local key informants) who could provide insights and feedback related to the intervention were identified. In each cluster, interviews or FGDs were conducted with all categories of informants we identified. The high-risk and non–high-risk cohort participants were selected based on their availability at the time of data collection.

Based on prior qualitative research experience, we planned a large volume of interviews and FGDs that were expected to yield sufficient informational redundancy. Data collection was completed as planned, followed by thematic analysis. During analysis, we observed substantial repetition of themes and no emergence of new insights relevant to the study objectives, indicating that the dataset was sufficient. Accordingly, no additional data collection was undertaken.

Data were collected within 2 months after the end of the intervention and before the trial results were known. In Haryana, the intervention ended in September 2021, and data collection was done (by AM, AK, and MD) in December 2021. In Andhra Pradesh, the intervention ended in December 2021, and data collection was done (by AM, S Kallakuri, and SD) in January 2022. All data were collected face-to-face. The data collection team consisted of 2 male and 4 female researchers with a PhD or a master’s degree and prior experience in collecting qualitative data. All members of the data collection team (except AM) were involved in training and oversight of trial activities but were not engaged in routine field-level implementation and had limited prior interaction with study communities. This partial insider position presented both constraints and opportunities. Familiarity with intervention logic and operational challenges supported contextually informed probing and interpretation of implementation experiences. At the same time, participants may have perceived researchers as institutionally affiliated outsiders, potentially encouraging socially desirable responses. However, community members and ASHAs openly articulated implementation challenges, including difficulty recalling IEC content and perceptions that repeated visits were unnecessary. In a small number of ASHA FGDs, the individual involved in delivering training also facilitated data collection; this dual role was recognized as potentially shaping responses and was considered reflexively during analysis. Data collection was intentionally gender-matched where feasible, with male researchers facilitating discussions with male community members, and female researchers conducting FGDs with female community members and ASHAs, to enhance comfort and openness, particularly among women participants.

Topic guides with probes were prepared in Hindi and Telugu. The objective of data collection was explained, and a participant information sheet was provided to all participants before starting the interview or FGD. Data were collected by team members proficient in the local language. In Andhra Pradesh, a few of the interviews and FDGs required translation support from a field supervisor, as one of the researchers (AM) could understand the local language but was not proficient in speaking it. Data were collected at clinics, community halls, and the homes of participants. All data were audio-recorded after taking consent from participants. The duration of FGDs ranged from 20 to 180 minutes. The interview duration ranged from 10 to 30 minutes. On average, 7 participants attended the FGD. The range was from 4 to 12. There were 2 FGDs with ASHAs that had only 4 participants. This was because in the selected PHC cluster, in all 6-7 ASHAs were part of the intervention, and when we conducted the FGDs with ASHAs, not all of them could attend.

A total of 33 interviews and 38 FGDs were conducted (Table 2). FGDs for men and women were done separately to honor social norms. A total of 288 participants (120 male and 168 female) were part of the qualitative study.

**Table 2:** Qualitative data sources.

Category		Andhra Pradesh (n=126), n	Haryana (n=127), n
**FGDs^a^**			
	ASHAs^b^	4	4
	High-risk women in the control arm	1	1
	High-risk men in the control arm	1	0
	High-risk women in the intervention arm	3	3
	High-risk men in the intervention arm	3	3
	Non–high-risk women in the intervention arm	3	3
	Non–high-risk men in the intervention arm	3	3
	Field staff	1	2
	Total	19	19
**Interviews**			
	PHC^c^ doctors	4	4
	Village head/community leaders	4	5
	High-risk individuals who did not seek care—intervention arm	6	4
	Government officials	3	3
	Total	17	16

^a^FGD: focus group discussion.

^b^ASHA: accredited social health activist.

^c^PHC: primary health center.

A detailed COREQ (Consolidated Criteria for Reporting Qualitative Research) checklist has been provided in Multimedia Appendix 1.

### Data Analysis

Data analysis was done using the framework analysis approach. This approach uses an existing framework consisting of key thematic areas of inquiry. Under each thematic area, there are codes (which loosely correspond to subthemes). The qualitative data is coded or indexed within these broad themes and codes. In the final analysis, the data for each theme is summarized.

The conceptual framework of the study (Table 1) informed the initial framework for analysis and guided the development of a set of a priori codes aligned with key areas of inquiry. Select transcripts were read by the research team members (MD, AM, S Kallakuri, and SKY) to come up with additional codes emerging from the data. The codebook developed after this process was used by the team to code the transcripts. The codebook was iteratively refined during the data coding process, and additions to the codebook were made (Multimedia Appendix 2). Disagreements were resolved through discussion. In case of a lack of consensus, the final decision was taken by the process evaluation lead (AM). All researchers had real-time access to the codebook and data files. The NVivo (version 12; Lumivero) collaboration server was used for data coding. For each code, the codebook included a clear definition specifying its meaning and illustrative examples of the type of data to be coded under that code. The codebook was stored on a shared drive accessible to all members of the research team, and any revisions were documented and visible to all coders. This process supported the consistent application of codes across transcripts.

The final analysis was done by the first author (AM), who synthesized coded data within each theme. The findings are organized under the RE-AIM domains. Qualitative data were intentionally combined with quantitative data to explain cluster-level differences. In addition, qualitative and quantitative data were used to explain variation in service uptake across different levels of care.

### Ethical Consideration

This study was approved by the ethics committees of the George Institute for Global Health India (009/2018) and All India Institute of Medical Sciences, New Delhi, India (IEC-315/01.06.2018, RP-58/2018, RP-16/2018). The study was approved by the Health Ministry’s Screening Committee, Indian Council of Medical Research. Written informed consent was obtained from all participants prior to the data collection. No compensation was provided to study participants for participating in the trial. All data management processes were compliant with national privacy law. Data were securely stored and analyzed on servers located at the George Institute India office.

## Results

### Reach

The program achieved a broad reach across geographically diverse rural settings in 2 states. In total, 9928 adults across 44 rural PHC clusters were enrolled, and 1697 people identified as high risk were allocated to the intervention arm. Of these, 1585 people were followed up at the end of 1 year to assess the outcomes [7]. Intervention components reached the majority of high-risk individuals in the intervention clusters. Table 3 presents cluster‑level follow‑up, and consultation indicators summarized as medians and IQRs across the 22 intervention clusters**.** The median proportion of high‑risk individuals who received at least 1 ASHA follow‑up was 98% (IQR 96.6%-100%), and the median number of ASHA visits per individual was 11.5 (IQR 6.2-19.0), with notable variation across clusters. A median of 87.6% (IQR 51.9-93.7) of high‑risk individuals were both seen by a PHC doctor and followed up by ASHAs at least 6 times.

**Table 3:** Cluster‑level patient follow‑up and primary health center (PHC) doctor consultations in intervention clusters, summarized as median (IQR).

Cluster-level characteristic	Cluster‑level value, median (IQR)
High‑risk individuals followed at least once by ASHAs^a^ (%)	98.0 (96.6-100.0)
Individuals who consulted a PHC doctor at least once (%)	93.2 (91.9-95.9)
High‑risk individuals followed up at least 6 times by ASHAs and seen by a PHC doctor (%)	87.6 (51.9-93.7)
Number of ASHA visits per high‑risk individual	11.5 (6.2-19.0)

^a^ASHA: accredited social health activist.

The antistigma component used multiple delivery methods and was implemented both individually for the study cohort, comprising high-risk and non–high-risk participants, and at the community level. Community meetings were organized in common village spaces, during which awareness videos were screened using a projector. These meetings were attended by members of the study cohort as well as community members who were not recruited into the study.

In addition, project field staff conducted regular home visits to high-risk participants to ensure exposure to antistigma materials, particularly for those who may not have attended community meetings. Printed materials were distributed across all villages within each intervention cluster.

Live drama performances were conducted in 1 village per intervention cluster, followed by interactive discussions with audience members. Villages with relatively larger populations and adequate space to conduct public performances were selected for these live shows. In remaining villages where live performances were not feasible due to logistical constraints and cost considerations, video recordings of the drama were screened, accompanied by facilitated discussions around the content.

Exposure of the study cohort to each antistigma campaign component was systematically tracked throughout the intervention period. Table 4 presents cluster-level exposure to individual antistigma campaign components among high-risk and non–high-risk participants, summarized as median percentages with IQRs across PHCs. Overall, the intervention achieved high exposure to most antistigma materials. Exposure to live drama was lower compared to other campaign modalities, due to the infeasibility of conducting live performances in every village; however, recorded drama clips were used to ensure broader reach. Across intervention clusters, a median of 84% (IQR 65.7-95.9) of the study cohort was exposed to at least 1 audiovisual campaign component.

**Table 4:** Cluster-level exposure to antistigma campaign components summarized as median percentage (IQR) across primary health centers (PHCs).

Types of antistigma materials and strategies	High-risk individuals exposed per PHC (%), median (IQR)	Non–high-risk individuals exposed per PHC (%), median (IQR)
Door-to-door campaign	96.8 (73.7-100)	95.1 (81.7-97.1)
Pamphlets	59.2 (4.3-94.4)	66.4 (5.1-94.3)
Posters	75.6 (54.3-92.7)	75.0 (54.3-86.5)
Flipbook	95.6 (92.5-98.4)	91.9 (88.9-97.2)
Calendar	97 (93-99.8)	92.7 (87.3-97.4)
Lived experience video of patient	97.1 (91.5-100)	95.9 (90.7-98.2)
Promotional video	97.5 (93.5-100)	95.0 (91.1-97.6)
Short video message by film actor	96.9 (86.6-100)	96.5 (91.9-98.6)
Short animation videos (marriage and school context)	97.6 (84.7-100)	96.9 (89.8-98.5)
Live drama	27.1 (8.9-41.6)	19.5 (6.7-37.5)
Recorded video of drama	93.9 (68.9-98.4)	87.9 (66.1-97)
Drama and all video components	84 (65.7-95.9)	85.4 (64.2-94.9)

Exposure and dosage metrics were tracked using backend data and program monitoring data.

### Effectiveness

Overall, the community-level screening and referral by ASHAs was seen as an effective way to ensure care for people with CMDs. A PHC doctor who organized regular weekly psychiatry clinics at his PHC told us that the footfall in his weekly clinic was lower than what was seen in village health camps conducted as part of the intervention.

Several patients were referred to psychiatric care from the district hospitals. Mental health professionals in these hospitals found the SMART Mental Health approach to be useful. Prior to the intervention, the psychiatrists at the district level received very few patients referred to them by PHC providers. Most came through self-referral. However, during the intervention, psychiatrists in the district hospitals found that many more people with CMDs were referred by the PHCs' doctors. The providers found that involving CHWs for screening, identifying high-risk people, and having a referral and follow-up mechanism in place were useful strategies to reach a larger number of patients in need of psychiatric care.

The government too has its programs and schemes.... They do not cover all individuals. Your program, however, surveyed, identified and treated each individual. It was more focused and so it was more effective.

Mental health professional at district hospital, key informant interview 6

A person suffering from mental health problems will always hesitate to express his/her problem. However, in your project, the ASHA workers conducted surveys and then identified people having such problems. This approach was very successful.

Mental health professional at district hospital, key informant interview 6

High-risk people who consulted a doctor and experienced improvement acknowledged the importance of consulting the primary care doctor. In some cases, the process of sharing problems with the doctor and receiving positive advice led to perceived improvement, and no medication was required.

**Participant:** The doctor’s words gave me immense relief. She told me to stop drinking.... I really liked it. The doctor told me many things. I could share all my problems with the doctor. The doctor’s advice gave me a lot of confidence. It felt good. I liked it.... I used to feel suicidal in the past....

**Interviewer:** What is your opinion about our program?

**Participant:** Before your program, I would feel so depressed. I had lost all interest in life. After your program, I felt so relieved and satisfied. Your colleagues would visit us often and talk to us.

High-risk participant, interview 8

After visiting the doctor, they looked happy. They were happy that the doctor spoke nicely to them and advised them to remain calm. They did not need any medicines.

ASHA, FGD-7

The antistigma campaign received mixed responses, with better recall for video content compared to print material. One of the factors behind poor recall could be that majority of the study cohort in both sites have education only up to the primary level, with a large proportion being nonliterate. We found that while participants did not always recall the exact message delivered by the antistigma campaign, they acknowledged that the program had provided them with new information. Participants said that it was important to seek help if someone was undergoing mental stress, and there were treatments available for mental illnesses.

They (project staff) came and showed a few photos and a few videos. One of the videos showed how a girl, who was suffering from some mental ailment, was cured with proper medication...they gave us some pamphlets, but I am uneducated. So, I did not read them.

High-risk participant, interview 7

**FGD Participant:** They (videos) were about mental health problems. The message was that if someone has a problem, he/she should not despair, because it can be treated.

Non–high-risk women, FGD-6

### Adoption

The SMART Mental Health platform, including the integrated EDSS, was viewed by doctors as a useful tool for the diagnosis and management of CMDs. None of the interviewed PHC doctors had previously used a decision support tool for mental health assessment. Doctors provided positive feedback on the availability of patient-specific diagnostic and follow-up information on the platform, which supported ongoing patient management.

There was no problem. The device was easy to operate. The questions were simple. The follow up procedure too was simple. All that we had to do was to enter the patient’s ID, and all the information would display on the screen.

Doctor, interview 6

We could diagnose if a person needed medication or whether that person just needed some counselling or if that person had some psychiatric illness with the help of the questionnaire.

Doctor, interview 7

The ASHAs used standardized tools—Patient Health Questionnaire-9 [12] and Generalized Anxiety Disorder-7 [13]—integrated in handheld tablets to screen people at high risk of depression, anxiety, and self-harm. The SMART Mental Health platform also provided them with a traffic light–based priority listing app to follow up patients. This was a novel experience for ASHAs as they had never used a digital device for community-level screening or follow-up. Some of them had never used a smartphone and initially were not confident about using a tablet device. ASHAs reported that good training and individual support to clarify their queries were crucial in helping them overcome initial reservations and use the mHealth platform with ease.

All those trainers who were there, they were all very good. They taught us to operate the tab so well.... Like me, I was not able to understand and ask the questions (in the tab). So, they explained it to me again and again. They gave special training to those who could not understand.

ASHAs, FGD-3

Because we had never used any smart device before, we found it difficult to use the tab in the beginning. We would often get confused. Later, though, we got over it. Our ‘sirs’ (referring to project staff) were excellent teachers.

ASHAs, FGD-5

Following initial support during the early phase of implementation, ASHAs reported feeling comfortable using the tool.

### Implementation

#### Contextual Influences on Cluster‑Level Variation

The intervention was implemented across 3 districts in 2 states—Andhra Pradesh and Haryana—which differ in language, cultural norms, and socioeconomic context. We reviewed outcome and reach indicators from the 8 clusters purposively selected for the process evaluation to identify cluster‑level variation and examine how contextual differences potentially shaped differences in implementation and outcomes.

While primary outcomes such as knowledge, attitudes, and stigma scores did not show marked differences across clusters, substantial variation was observed in self‑reported receipt of mental health care (Table 5). Variation was also observed in the mean number of ASHA visits across states, with clusters in Andhra Pradesh showing higher average visit counts than those in Haryana (Table 5). In the table, clusters 1-4 were in Haryana, and clusters 5-8 were in Andhra Pradesh.

**Table 5:** Accredited social health activist (ASHA) follow‑up, primary health center (PHC) doctor consultations, and self-reported receipt of care in intervention clusters selected for process evaluation (descriptive statistics).

Cluster name	High-risk participants in each cluster, n	High-risk participants followed up by ASHAs at least once, n (%)	High-risk participants who consulted a PHC doctor at least once, n (%)	High-risk participants followed up by ASHAs at least 6 times and seen by doctor, n (%)	Visits by an ASHA per high-risk individual, mean (SD)	Self-reported receipt of mental health care, n (%)
Cluster 1 (intervention)	176	170 (96.6)	100 (56.8)	93 (52.8)	8 (3)	135 (76.7)
Cluster 2 (intervention)	230	227 (98.7)	213 (92.6)	47 (20.4)	4 (2)	199 (86.5)
Cluster 3 (intervention)	118	114 (96.6)	113 (95.8)	51 (43.2)	6 (3)	102 (86.4)
Cluster 4 (intervention)	140	N/A^a^	N/A	N/A	N/A	47 (33.6)
Cluster 5 (intervention)	44	43 (97.7)	41 (93.2)	41 (93.2)	19 (5)	36 (81.8)
Cluster 6 (intervention)	53	52 (98.1)	48 (90.6)	47 (88.7)	16 (6)	47 (88.7)
Cluster 7 (intervention)	36	36 (100)	36 (100)	36 (100)	21 (3)	36 (100)
Cluster 8 (control)	77	N/A	N/A	N/A	N/A	2 (2.6)

^a^N/A: not applicable.

Cluster 1 had a markedly lower proportion of participants who had consulted a PHC doctor at least once. Self‑reported receipt of mental health care was broadly similar across intervention clusters 2, 3, 5, and 6, but was lower in cluster 1 and highest in cluster 7. Self‑reported receipt of mental health care was also substantially higher in the Haryana control cluster (cluster 4) than in the Andhra Pradesh control cluster (cluster 8). Mean ASHA visits were higher in Andhra Pradesh than in Haryana.

Qualitative findings suggested several contextual factors underlying these differences. Intervention cluster 1 included a highly transient truck driver population, limiting consistent availability, and delayed village health camps due to PHC doctor deputation. In Andhra Pradesh, field teams proactively monitored backend data and prompted ASHAs to follow up patients at regular intervals, ensuring higher mean follow-ups. In Haryana, the field teams adopted a less intensive nudge strategy.

Differences between the 2 control clusters also appeared to reflect contextual variation. In the Haryana control cluster (cluster 4), ASHAs were described as receptive and proactive; they maintained regular contact with project field staff and referred several cases to the district hospital. In contrast, the control cluster in Andhra Pradesh (cluster 8) was a large village located near a periurban area, with many households reporting relatively higher incomes than the local ASHAs. Community interaction with ASHAs and the public health system was limited, as families preferred to seek care from private providers in a nearby town. Although ASHAs communicated the need to seek care to high‑risk families, these households showed limited interest in visiting the PHC.

#### Care Seeking Mental Health

People identified at high risk of depression, anxiety, or self-harm were encouraged to consult a doctor. A majority (1431/1697, 84.3%) consulted the PHC doctor either at the PHC or at a health camp organized in their village. However, fewer than a quarter of patients referred to the psychiatrist did actually consult them. The numbers were higher in Andhra Pradesh (43/122, 35.2%) compared to Haryana (10/102, 9.8%; Table 6).

**Table 6:** Care seeking by the “high-risk” cohort.

	Andhra Pradesh (n=444), n (%)	Haryana (n=1253), n (%)	Total (n=1697), n (%)
Total patients seen at PHC^a^	143 (32)	452 (36.1)	595 (35.1)
Total patients seen at health camp	184 (41)	653 (52.1)	836 (49.3)
Total patients referred to psychiatrist	122 (27.4)	102 (8.2)	224 (13.2)
Total patients seen by psychiatrist (out of total referred)	43 (35.2)	10 (9.8)	53 (23.6)

^a^PHC: primary health center.

The most common reason stated was related to physical accessibility and the costs associated with visiting the health center.

The reason they cite is that they do not know the place. If we make arrangements, they will go. They are unwilling to spend money for auto charges. In case I arrange for a vehicle to take them, they will come up with some excuse or the other not to go. A few do go though. I sent two of them on a bike to _____ PHC. This I did twice.

ASHA, FGD-5

I have money problem; I am very poor. There is no help. I have a son who struggles to take care of his kids and me. What do I do now?

High-risk participant, interview 3

Some older people and women reported the lack of a companion to accompany them to the hospital as a further barrier. Personal preferences also influenced care seeking. Some felt that they were not unwell and did not need medical advice. Others felt they were not yet ready to seek help.

Fear of being perceived negatively by others due to the stigma associated with mental health was a barrier in some cases.

**Interviewer:** But what is the reason for not going (to the doctor)?

**Participant 1:** The fear that everyone would come to know about them is the reason. They thought it was a matter of losing face in society.

**Participant 2:** Yes. They feared that people would look upon them as outcasts if they came to know about them.

**Interviewer:** Is there any other reason?

**Participant 1:**That is the only reason; that what the people will think of them.

ASHA, FGD-5

Regular follow-up by ASHAs was an important facilitator that helped high-risk individuals seek care. Patients who made the decision to visit the doctor despite their initial hesitation mentioned the role of the ASHAs. In a few cases, ASHA workers were proactive in arranging for transportation to the PHC.

I used to think that there can be no treatment, but she (ASHA) kept saying, that at least get it checked once (by a doctor).... She spoke to me three or four times.... I have no regrets now.

High-risk participant, interview 1

She (the ASHA) is good. She is genuinely concerned (for our wellbeing) and speaks very warmly. She tries to convince us to visit the PHC if we are unwell. She instils a lot of courage in us.

High-risk participant, interview 6

Making services more accessible was another strategy that facilitated care seeking. Health camps were organized in villages that had poor connectivity with the PHC. The PHC doctor visited the village on a specific day and conducted an outpatient clinic. ASHAs and project staff informed and motivated high-risk people to visit the doctor during the camp. This approach was successful in improving coverage.

It is because of the camp that so many people came. That’s because neither did they have to pay for conveyance, nor did they have to go out somewhere. So they got it done (themselves checked) on their own.

ASHA, FGD-2

Another strategy to improve access was teleconsultation. This was feasible only in Andhra Pradesh and was implemented because of COVID-19–related travel restrictions set by the government. At an appointed time, the patient consulted the psychiatrist associated with the District Mental Health Program using tablets. The staff was present to help facilitate the call. The consultation was done in private in the homes of high-risk people. Patients appreciated the ease of access to a doctor from their homes.

I felt very nice that I could tell the doctor all my problems without having to go anywhere.

High-risk men, FGD-6

While overall there was a good response to teleconsultation, some doctors had reservations regarding the method, as they felt face-to-face consultations led to better patient interaction and satisfaction.

#### Barriers and Facilitators in Implementation

Implementation of the antistigma campaign had several challenges. Sustaining interest in the study cohort in the antistigma content over the entire intervention period was difficult. While there was a lot of interest in the awareness videos at the start of the intervention, in the later phase, some patients found it repetitive and did not show interest in watching the videos. Several patients found repeated visits by project staff unnecessary.

Whatever videos we were showing them (the study cohort), all those videos should be shown to one person at the same time once only. We had shown them to the same patient four times. Some day we were showing them one video, some other day we were showing them some other video. So they were getting irritated and said that ‘why you are coming again and again?’ This was observed in both high-risk as well as non–high-risk patients.

Field staff, FGD-1

There were some cases of outright refusal to further engage with the research team. Some of this could be attributed to stigma and worry about other community members’ perceptions. Since the research required intensive engagement with the cohort, some felt being singled out for a mental health intervention would raise questions among neighbors.

We did not mind (project staff coming often). But our neighbors would keep asking us as to why they would come so often. This interference was quite irritating.... One of my neighbors would keep bothering me with her questions.

Non–high-risk women, FGD-6

Time and the availability of participants were important factors. Some villages consisted of migrant laborers who were only available seasonally. In other villages, the nature of work (trucking and fishing) required long engagement, making it necessary to remain available outside working hours. The staff and ASHAs had to make additional efforts to reach out to such people.

We faced a lot of problems too ma’am. I have done screening at 8 or 9 at night. People weren’t available during the day.

ASHA, FGD-1

Cultural barriers were also encountered in a few villages that affected the overall participation of the study participants. According to local cultural norms in Haryana, Muslim women were not expected to watch videos or interact with men alone (in this case, the men they are referring to are the field staff who used to go to show the IEC materials).

The men used to say that- ‘brother, don’t come and show such IEC material. Our religion is not such that some men come from outside sit near our women and show them content in this way. It’s not in our religion to watch such videos.

Field staff, FGD-1

To address this, staff would make sure that male family members were present during interactions. ASHAs, who were local women, were given a more prominent role in showing antistigma material in such villages. Efforts were also made to explain the informational nature of the videos. When required, endorsement was taken from the local religious heads.

The COVID-19 pandemic was an important further barrier to implementation. India experienced several waves, including a very severe second wave in 2021. It was a challenge to conduct research activities and maintain regular interaction with the study cohort at this time. Staff and ASHAs were refused entry into households due to fear of infection.

In...PHC (area), people did not open the door at all. Due to COVID, they did not open their doors, no matter how long we stood (outside).

Field staff, FGD-1

**Participant 1:** They told us not to come because they were scared that they too would get infected....

**Participant 2:** A few of them would say that you people move around the village. You might be the carriers.

**Participant 4:**They said that because we have dealings with COVID patients, we should not visit their houses.

ASHA, FGD 7

Another problem faced in a few villages was related to a poor telecommunications network and connectivity, which impacted the real-time upload of data. Most villages have connectivity for mobile and internet services; however, depending on the service provider, signal strengths would vary. In such villages, locations with relatively good signal strength were identified. Our staff was provided with dongles with SIM cards from different providers. In case any ASHA faced difficulty in uploading data, our staff assisted with uploading the data.

The antistigma campaign used a combination of print content, audiovisual content, and live performance. The acceptability of audiovisual content was higher compared to printed information (pamphlets and brochures). The live drama received extremely positive feedback in Andhra Pradesh, whereas in Haryana, it got a mixed response. The cultural context played an important role in this. In Haryana, public stage performances at the village level are usually for the entertainment of men, with a few women attending. A theater performance was a novel concept, and women had to be especially invited to attend the event. In 1 village in Haryana, the show had to be wrapped up early due to unruly crowds.

Implementation of SMART Mental Health required close interaction and buy-in from ASHAs, PHC doctors, and the community. For the ASHAs, regular interactions, handholding, support, and critical feedback when needed were important factors that facilitated active participation in project activities. Additional handholding and support were extremely important for ASHAs who had not used digital platforms earlier. Some of them had never used smartphones, and this was a novel experience for them.

The staff that you sent; they were all good; without them we wouldn’t have succeeded (in completing project activities) If we needed their help ten times, they would come help us ten times.

ASHA, FGD-1

Regular interactions were also maintained with PHC doctors. Implementation staff were present to provide any assistance with the EDSS platform. Formal permission from the state-level health department was another factor that was important in gaining cooperation from PHC doctors.

Initial buy-in from the community was an important facilitator. There was mistrust of outsiders and nongovernment organizations especially in Haryana. Employing local staff who spoke the local language and dialect and belonged to the area helped in addressing some of the suspicions.

### Maintenance

The PHC doctors felt that, given the low levels of training in psychiatry, they usually lacked the ability to diagnose and manage CMDs. However, capacity building, combined with the use of the EDSS, had provided them with the skills to diagnose and manage people with CMDs. One positive impact was that some of them had started asking for mental health–related questions in their regular history taking during outpatient clinics, even with patients not part of the intervention, something they had not done earlier.

I have patients in the OPD (Out-patient department). Now I also try to check if the patient is a mental health related patient, if there is any need for a psychiatric opinion in that case or not... we ask if the patient is getting any negative thoughts in the mind, is he/she able to sleep properly, does he/she have insomnia or memory loss. Such questions have increased now.

Doctor, interview-1

The ASHAs provided positive feedback about transitioning to a digital system for recording data and patient follow-up. They felt that the skills they had learnt in the project were useful, given the government’s push on digitalization. There was also acknowledgment that the training had helped to ASHAs to gain skills in identifying people with mental health care needs in the community and that this skill would be useful for them in any future programs on mental health.

We learnt how to use a tab. That is something to be happy about. Today, all of our work is online, and still because we know how to use tab, there is no issue left. Even though we are literate but honestly, we didn’t even know how to use phone properly. We just have phone for calling and talking, nothing else.

ASHA, FGD-1

First of all, we learned to operate tab and then the phone. Secondly, we came to know how many people in our community are dealing with mental health issues.... Now if we get a chance to work on a similar project, we can handle it easily as we have experience of this work. We can handle it more easily and perfectly. We can identify such people very easily who are under depression.

ASHA, FGD-3

## Discussion

The results helped us to understand the key facilitators and barriers in the implementation of a complex intervention such as SMART Mental Health. Based on the MRC guidelines, we focused on “context” and “implementation” as broad areas of inquiry and suggest here a plausible “mechanism of change.”

### Context: Intervention Setting and Its Interaction With the Implementation

Context can refer to a range of factors that affect or can be affected by the intervention, including but not limited to social, cultural, organizational, or policy-related factors. Identifying and reporting relevant contextual factors that led to adaptations or impacted outcomes can be useful for designing and scaling up similar interventions [14,15].

The local context presented several cultural barriers. Efforts were made to address concerns of Muslim families in Haryana regarding audiovisual content and interaction with male staff. Similarly, additional efforts were made to reach out to groups that are difficult to reach, such as migrants, the fishing and truck driver communities. Our experience shows that additional time, planning, and resources are needed to ensure vulnerable groups are included in a mental health intervention.

COVID-19 led to certain adaptations, such as introducing teleconsultation in Andhra Pradesh. The program also needed to adapt and put in place strategies that could address barriers to accessing care at the PHC due to a lack of transport and the cost of travel. Bringing PHC doctors to villages to conduct health camps was helpful. These adaptations were made based on what was feasible and acceptable during implementation, rather than following a predefined adaptation framework, in order to respond to contextual constraints. While core intervention components—such as screening and clinical decision support—remained standardized, delivery strategies were adapted to local conditions. Similar context-specific adaptations, such as adjusting meeting times and conducting health camps for difficult-to-reach communities in the vicinity of these communities, have been highlighted as a useful strategy by health functionaries in another state in India [16].

Stigma, particularly perceived stigma, was a barrier in seeking care that was highlighted during formative work and by other research in the study area [3,4,17]. The antistigma campaign used previously piloted as well as newly developed evidence-based strategies in the community [17-19]. The study found that the audiovisual medium was more acceptable compared to printed content. The intervention ensured that the high-risk cohort was exposed to the content multiple times. This was, however, a less acceptable strategy and did not increase recall. In most cases, the videos were shown individually. Showing the material in small community groups can be an alternate strategy. This will ensure that people do not feel singled out for mental health messaging. It can also provide opportunities for group interaction and engagement.

### Implementation: Reach, Effectiveness, Adoption, and Maintenance

The coverage or ‘reach’ of the intervention was ensured by addressing key barriers discussed earlier. Exposure to the content of the antistigma campaign and follow-up of high-risk cohort by ASHAs was high. This was achieved through meticulous monitoring of individual-level data and planning follow-up visits and patient consultation with ASHAs and doctors to ensure all high-risk people were reached. Program‑monitored data provided a reliable measure of exposure and follow‑up, capturing coverage rather than the depth or quality of engagement, which was examined through qualitative analysis. While reach is a core component of the RE‑AIM framework, it does not capture heterogeneity in engagement among those reached. Maximizing reach required intensive delivery and repeated exposure to intervention components; however, this intensity was not uniformly beneficial. Qualitative findings indicate that repeated exposure to antistigma content led to diminishing returns for some participants, including irritation and limited recall, suggesting potential saturation effects. Together, these findings highlight a key tension between maximizing coverage and sustaining engagement and underscore the importance of considering both dose and quality of delivery when interpreting implementation fidelity and success.

The “effectiveness” of an intervention refers to the extent to which an intervention achieves its intended outcome. This has been discussed in detailed trial results [7]. The process evaluation captured perceptions of stakeholders on why they felt the intervention was effective or not. A community-based screening model for CMDs was perceived as an effective strategy by PHC doctors as well as specialist mental health professionals providing care at the district hospital. Doctors at PHCs rarely diagnosed or referred patients with CMDs prior to the intervention. Limited training and experience in diagnosing symptoms of CMDs were reasons cited by PHC doctors. The training, EDSS, and consultation with high-risk patients helped to address this gap. There was an increase in the number of patients referred by PHC doctors in the intervention to the district psychiatrist.

“Adoption” to the extent to which users and implementers are willing to take up the intervention. We found that the digital component was a novel experience for both ASHAs and doctors. The EDSS was seen as a useful tool for diagnosis and treatment by PHC doctors. Our findings show that to enable the adoption of digital health interventions among CHWs and doctors, 1-time training is not enough. It needs to be supplemented with continuous handholding, support, and feedback in the early implementation phase. Some intangible aspects, such as good rapport building and respectful communication by project staff, were important in ensuring buy-in from CHWs. Other studies in low- and middle-income countries (LMICs) contexts have highlighted the importance of understanding the variation in experience, education, and exposure to digital technologies among CHWs before starting the training. Effective training of CHWs has been found to be a key element in successful mHealth interventions [20].

There were several positive spillover effects that ASHAs reported. Many felt that they had personally benefited from participating in SMART Mental Health. Some ASHAs felt that they had overcome the inhibition of speaking with male members in the community. Others reported that improved understanding of mental health and the practice of asking questions during screening had provided them with an important skill. It had helped them to connect better with the community, to listen to them and discuss their problems.

Within the RE‑AIM framework, “maintenance” is conceptualized as the long‑term sustainment of intervention effects and continued delivery of intervention components beyond the active trial period. Direct assessment of posttrial maintenance lies beyond the scope of this process evaluation. A separate study conducted 3 months into the posttrial phase specifically examines issues related to intervention maintenance and will be reported elsewhere. However, this process evaluation provides important insights into early indicators and conditions likely to influence maintenance, particularly in the context of scale‑up within the public health system.

One such area was related to human resource training. We found that intensive handholding support, including village-level supervision in the initial months, was an important facilitator for the adoption of the EDSS for the ASHAs. While scaling up such interventions, legitimate concerns related to who can provide this support, how much time and resources are needed, and whether it is sustainable in the existing health system need to be thought through. Midlevel health workers with whom ASHAs have a direct reporting relationship can be trained to provide this initial handholding and supportive supervision, as it complements their existing responsibilities.

ASHAs perceived increased community respect and recognition of their role as health workers and attributed it to technology use and its association with competence and credibility. With the government introducing digital data systems at the same time, ASHAs in the project saw themselves as early adopters and better prepared than their peers. The alignment of the intervention with government digital initiatives suggests strong maintenance potential. ASHA’s perceptions of increased preparedness and relevance indicate that core intervention components were compatible with routine system requirements, supporting the likelihood of continued use beyond the intervention period. At the end of the intervention, the PHC doctors were provided with a hard copy of the questions used in the EDSS for future reference. Doctors reported including questions related to mental health in their regular history taking for patients, indicating partial maintenance of some clinical practices even in the absence of the EDSS.

### Mechanism of Change: Potential Explanatory Pathways

Mechanism of change refers to a theory of why change occurs or how an intervention leads to particular outcomes. This is of relevance for scaling and designing future programs. The assumptions and causal mechanisms articulated at the design stage may evolve as we accumulate more evidence on outcomes, implementation processes, and context.

SMART Mental Health articulated several hypothesized pathways to change at inception. Stigma is a major demand-side barrier for mental health help-seeking; hence, it was theorized that the antistigma campaign would improve knowledge and attitudes related to mental health stigma, thereby increasing demand for mental health services. In parallel, the EDSS combined with training of PHC doctors and ASHAs was intended to improve their capacity to identify, treat, and refer individuals at high-risk of CMDs.

Our results show clear improvements in stigma‑related knowledge, attitudes, and perceptions about help‑seeking. However, these improvements did not translate into corresponding changes in stigma‑related behavior. Consistent with the stigma literature, behavioral outcomes are typically less sensitive to intervention effects than knowledge or attitudes, with several reviews reporting smaller and less consistent effects on stigma‑related behaviors even when attitudinal outcomes improve. Similar patterns have been seen in outcomes related to mental health help-seeking behavior [21-23]. This signals the well‑recognized *intention-behavior gap*, where positive attitudes and intentions do not always lead to action [24]. Literature to explain the intention-behavior gap has often highlighted individual-level factors, such as the role of intentions, attitudes, subjective norms, or perceived behavioral control, and, to a lesser extent, highlighted possible structural factors such as access to services [25,26]. Other factors that may explain this gap include temporal lag. Behavior change may take longer to emerge than attitudinal change, and short follow‑up periods may fail to capture delayed behavioral effects [24,27]. Notably, despite low stigma‑related behavioral scores, a substantial proportion of individuals in the intervention arm consulted primary care providers, suggesting that the intervention was successful in increasing demand for mental health care at the PHC level. In contrast, a markedly smaller proportion of individuals attended specialist services following referral. This steep attrition along the referral cascade represents one of the most policy‑relevant findings of the process evaluation and points to structural supply‑side constraints that may cap the impact of primary care digital interventions, a pattern widely documented in global mental health task‑sharing initiatives [28,29]. While the intervention actively reduced access barriers to PHC-based care by facilitating village-level visits by PHC doctors along with follow-up support by ASHAs, similar facilitation was not available for specialist care. Barriers such as distance to facilities, travel and treatment costs, and limited availability or perceived quality of specialist services reduced referral completion. This reflects broader evidence from LMICs showing that weaknesses in the health system often outweigh individual-level changes in shaping mental health service use [28].

Differences between states further highlight the importance of the health-system context. In Andhra Pradesh, where district mental health services were relatively better established and the psychiatrist was mandated by the local health administration to make regular visits to PHCs, a higher proportion of referred individuals accessed specialist care compared to Haryana, where the psychiatrist was unable to make such visits due to a high patient load at the hospital and a vacant post for an additional psychiatrist. This suggests that the benefits of primary care digital interventions may be capped by supply-side constraints such as limited specialist availability, high clinical workloads, and weak referral systems. This is consistent with findings from global reviews of primary mental health care integration [29].

Beyond formal service use, participation in the intervention appeared to enable relational and supportive processes that may have contributed to symptom improvement. Qualitative accounts indicate that high‑risk individuals valued opportunities to share their concerns with PHC doctors, ASHAs, and project staff. Regular follow-ups often involved asking about the well-being of the high-risk individuals. Some ASHAs took an interest in spending time and listening to the problems of the high-risk people in their community. Drawing on counseling and psychotherapy literature as an interpretive lens, processes such as feeling heard, understood, and accepted are widely discussed as potentially therapeutic. We invoke this literature to help interpret participants’ experiences, rather than as confirmatory evidence of effect, and to suggest that these relational interactions may represent an additional, unanticipated mechanism through which the intervention could have supported symptom improvement [30].

EDSS was perceived as an important aid to reach a diagnosis by PHC doctors. It was, however, emphasized that individuals with mental health–related problems needed separate dedicated clinic timings as more time was needed for taking history, making the patient comfortable about discussing their problems, and counseling them, which was difficult to provide in the PHC setup. Another challenge related to making psychotropic medications available at the PHCs was also recognized by the PHC doctors. Due to limited demand in the past, these medicines were rarely indicated at the PHCs. Some doctors preferred to refrain from prescribing psychotropic medications themselves due to fear of possible side effects and referred any patients in need of medication to the psychiatrist. Together, these findings underscore the complexity of mechanisms underpinning the intervention: technological decision support alone was insufficient without concurrent alignment of clinical time, medication supply, provider confidence, and referral systems.

### Limitations

The data collection for the process evaluation was done by researchers who were purposively blinded to results. This was done to reduce possible bias resulting from knowledge of results. However, a disadvantage of this was that specific questions that explain some of the results could not be asked during data collection. For example, we could not specifically enquire why improvement in knowledge and attitude related to stigma did not lead to a change in behavior.

The process evaluation relied largely on self-reported data from participants and providers, which may be subject to social desirability bias. Some respondents may have overreported positive attitudes, knowledge, or perceptions about the intervention.

In our protocol [9], we had planned to capture detailed differences in some key metrics across the clusters. However, some of the data, such as time taken to administer the screening tool by ASHAs and the EDSS tool by doctors, could not be used in this paper due to difficulty in authenticating the actual start time and end times taken during administration of the tools due to technical issues in the design of the tools. In some cases, the app was not switched off till much after the end of the patient consultation. In other cases, doctors asked questions from memory first and consulted the tablet later. Similar challenges were faced by ASHAs.

The process evaluation was conducted in a specific context with particular levels of health system constraints, human resource capacities, and digital readiness. Therefore, these findings should not be generalized to other LMICs without due consideration to the local context.

### Implications for Future Research and Policy

The findings from the SMART Mental Health trial have important implications for mental health–related service delivery in India and other LMIC settings. The intervention was delivered with the existing cadre of primary health workers, including PHC doctors and ASHAs. It demonstrated the ability of primary care doctors to integrate EDSS in their practice with the right training and support. The Indian government has put forth a plan for comprehensive primary health care in which mental, neurological, and substance use disorders will be integrated into primary care [31]; entitled, the national mental health policy of the Government of India [32], which also aligns with the World Health Organization’s Comprehensive Mental Health Action Plan 2013-30 [33]. A system for community-level screening for CMDs by ASHAs, as well as referral and management of CMDs are part of these policy guidelines. The policy envisages patient-level digital health records at the primary care level, but leaves much scope to explore possibilities of integrating digital technology. There is potential for service delivery using digital platforms. The SMART Mental Health platform has been successfully used to manage referrals, ensure follow-up, and integrate an EDSS for improved diagnosis and management of mental, neurological, and substance use disorders. Any such initiative needs to ensure adequate support for training, supervision, and incentives from health care workers to ensure sustainability. There is also a need to ensure that the overall digital infrastructure is strengthened, particularly in remote regions, to avoid inequities in access to digital services. Future research should focus on scaling this model and further research to understand how improved knowledge and attitudes related to mental health stigma can translate to positive behavior change.

## Appendix

Multimedia Appendix 1 COREQ checklist for SMART mental health process evaluation.

Multimedia Appendix 2 Codebook for data analysis.

## Abbreviations

ASHA: accredited social health activist
CHW: community health worker
CMD: common mental disorder
COREQ: Consolidated Criteria for Reporting Qualitative Research
EDSS: electronic decision support system
FGD: focus group discussion
IEC: information, education, and communication
LMIC: low- and middle-income country
mHealth: mobile health
MRC: UK Medical Research Council
PHC: primary health center
RE-AIM: Reach, Effectiveness, Adoption, Implementation, and Maintenance
SMART: Systematic Medical Appraisal, Referral, and Treatment
