# Correction: Barriers and Facilitators in the Implementation of the Systematic Medical Appraisal, Referral, and Treatment (SMART) Mental Health Digital Intervention in Rural India: Mixed Methods Process Evaluation Study

**DOI:** 10.2196/101441

**Published:** 2026-06-12

**Authors:** Ankita Mukherjee, Mercian Daniel, Sudha Kallakuri, Siddhardha Devarapalli, Sandhya Kanaka Yatirajula, Amanpreet Kaur, Praveen Devarsetty, Usha Raman, Beverley M Essue, Rajesh Sagar, Shashi Kant, Shekhar Saxena, Graham Thornicroft, Anushka Patel, David Peiris, Pallab K Maulik

**Affiliations:** 1 George Institute for Global Health New Delhi, Delhi India; 2 George Institute for Global Health Hyderabad, Telangana India; 3 Jindal School of Psychology & Counselling OP Jindal Global University Sonipat, Haryana India; 4 Faculty of Medicine and Health University of New South Wales Sydney, New South Wales Australia; 5 Prasanna School of Public Health Manipal Academy of Higher Education Manipal, Karnataka India; 6 Department of Communication University of Hyderabad Hyderabad, Telangana India; 7 Institute of Health Policy, Management and Evaluation University of Toronto Toronto, ON Canada; 8 Department of Psychiatry All India Institute of Medical Sciences New Delhi, Delhi India; 9 Department of Community Medicine All India Institute of Medical Sciences New Delhi, Delhi India; 10 Harvard T H Chan School of Public Health Harvard University Boston, MA United States; 11 Institute of Psychiatry, Psychology and Neuroscience King's College London London, England United Kingdom; 12 The George Institute for Global Health Sydney, New South Wales Australia

In “Barriers and Facilitators in the Implementation of the Systematic Medical Appraisal, Referral, and Treatment (SMART) Mental Health Digital Intervention in Rural India: Mixed Methods Process Evaluation” [1], the author has made one correction. One author’s name was inadvertently deleted from the metadata in the final proof. (His name was there in all previous versions of the manuscript.)

The author list was revised from the following:

Ankita Mukherjee^1^, PhD; Mercian Daniel^1^, PhD; Sudha Kallakuri^2^, PhD; Siddhardha Devarapalli^2^, PhD; Sandhya Kanaka Yatirajula^1^, PhD; Amanpreet Kaur^1,3^, PhD; Praveen Devarsetty^2,4,5^, MBBS, MD, PhD; Usha Raman^6^, PhD; Beverley M Essue^7^, PhD; Rajesh Sagar^8^, MBBS, MD; Shashi Kant^9^, MBBS, MD; Shekhar Saxena^10^, MD; Graham Thornicroft^11^, MBBS, MSc, PhD; Anushka Patel^4,12^, MBBS, SM, PhD; Pallab K Maulik ^1,4,5^, MSc, MD, PhD

The corrected author list now reads:

Ankita Mukherjee^1^, PhD; Mercian Daniel^1^, PhD; Sudha Kallakuri^2^, PhD; Siddhardha Devarapalli^2^, PhD; Sandhya Kanaka Yatirajula^1^, PhD; Amanpreet Kaur^1,3^, PhD; Praveen Devarsetty^2,4,5^, MBBS, MD, PhD; Usha Raman^6^, PhD; Beverley M Essue^7^, PhD; Rajesh Sagar^8^, MBBS, MD; Shashi Kant^9^, MBBS, MD; Shekhar Saxena^10^, MD; Graham Thornicroft^11^, MBBS, MSc, PhD; Anushka Patel^4,12^, MBBS, SM, PhD; David Peiris^4,12^, MIPH, PhD; Pallab K Maulik^1,4,5^, MSc, MD, PhD

The correction will appear in the online version of the paper on the JMIR Publications website, together with the publication of this correction notice. Because this was made after submission to PubMed, PubMed Central, and other full-text repositories, the corrected article has also been resubmitted to those repositories.
