# Supplementary material for: Barriers and Facilitators in the Implementation of the Systematic Medical Appraisal, Referral, and Treatment (SMART) Mental Health Digital Intervention in Rural India: Mixed Methods Process Evaluation Study
Source: JMIR Ment Health. 2026 May 7;13:e89164. doi: 10.2196/89164 (PMC13195372; doi:10.2196/89164)
Supplement: Multimedia Appendix 1 [file mental_v13i1e89164_app1.docx]

**COREQ (COnsolidated criteria for REporting Qualitative research) Checklist**

Title: **Barriers and Facilitators in implementation of a digital mental health intervention in rural India: Findings from the Process Evaluation of the SMART Mental Health cRCT**

| **Topic** | **Item No.** | | **Guide Questions/Description** |  | | **Reported on**  **Page No.** |
| --- | --- | --- | --- | --- | --- | --- |
| **Domain 1: Research team and reﬂexivity** | | | | | | |
| *Personal characteristics* | | | | | | |
| Interviewer/facilitator | 1 | | Which author/s conducted the interview or focus group? |  | | Page 7 |
| Credentials | 2 | | What were the researcher’s credentials? E.g. PhD, MD |  | | Page 7 |
| Occupation | 3 | | What was their occupation at the time of the study? |  | | Page 7 |
| Gender | 4 | | Was the researcher male or female? |  | | Page 7 |
| Experience and training | 5 | | What experience or training did the researcher have? |  | | Page 7 |
| *Relationship with participants* | | | | | | |
| Relationship established | 6 | | Was a relationship established prior to study commencement? |  | | No |
| Participant knowledge of the interviewer | 7 | | What did the participants know about the researcher? e.g. personal goals, reasons for doing the research |  | | Page 7 |
| Interviewer characteristics | 8 | | What characteristics were reported about the inter viewer/facilitator?  e.g. Bias, assumptions, reasons and interests in the research topic |  | | Page 7 |
| **Domain 2: Study design** | | | | | | |
| *Theoretical framework* | | | | | | |
| Methodological orientation and Theory | 9 | | What methodological orientation was stated to underpin the study? e.g. grounded theory, discourse analysis, ethnography, phenomenology,  content analysis |  | | Page 8 |
| *Participant selection* | | | | | | |
| Sampling | 10 | | How were participants selected? e.g. purposive, convenience, consecutive, snowball |  | | Page 6 |
| Method of approach | 11 | | How were participants approached? e.g. face-to-face, telephone, mail, email |  | | Page 7 |
| Sample size | 12 | | How many participants were in the study? |  | | Page 7 |
| Non-participation | 13 | | How many people refused to participate or dropped out? Reasons? |  | | None |
| *Setting* | | | | | | |
| Setting of data collection | 14 | | Where was the data collected? e.g. home, clinic, workplace |  | | Page 7 |
| Presence of non-  participants | 15 | | Was anyone else present besides the participants and researchers? |  | | Page 7 |
| Description of sample | 16 | | What are the important characteristics of the sample? e.g. demographic  data, date |  | | Page 7 |
| *Data collection* | | | | | | |
| Interview guide | 17 | | Were questions, prompts, guides provided by the authors? Was it pilot tested? |  | | Page 7 |
| Repeat interviews | 18 | | Were repeat interviews carried out? If yes, how many? |  | | No |
| Audio/visual recording | 19 | | Did the research use audio or visual recording to collect the data? |  | | Page 7 |
| Field notes | 20 | | Were ﬁeld notes made during and/or after the interview or focus group? |  | | No |
| Duration | 21 | | What was the duration of the interviews or focus group? |  | | Page 7 |
| Data saturation | 22 | | Was data saturation discussed? |  | | Page 6-7 |
| Transcripts returned | 23 | | Were transcripts returned to participants for comment and/or correction? |  | | No |
| **Domain 3: analysis and ﬁndings** | | | | | | |
| *Data analysis* | | | | | | |
| Number of data coders | | 24 | How many data coders coded the data? | |  | Page 8 |
| Description of the coding  tree | | 25 | Did authors provide a description of the coding tree? | |  | Supplementary file |
| Derivation of themes | | 26 | Were themes identiﬁed in advance or derived from the data? | |  | Advance-using REAIM |
| Software | | 27 | What software, if applicable, was used to manage the data? | |  | N-Vivo 12 |
| Participant checking | | 28 | Did participants provide feedback on the ﬁndings? | |  | No |
| *Reporting* | |  |  | |  |  |
| Quotations presented | | 29 | Were participant quotations presented to illustrate the themes/ﬁndings?  Was each quotation identiﬁed? e.g. participant number | |  | Yes, Page 11-20 |
| Data and ﬁndings consistent | | 30 | Was there consistency between the data presented and the ﬁndings? | |  | Yes |
| Clarity of major themes | | 31 | Were major themes clearly presented in the ﬁndings? | |  | Yes, Page 9-20 |
| Clarity of minor themes | | 32 | Is there a description of diverse cases or discussion of minor themes? | |  | Yes, Page 9-20 |

Tong A, Sainsbury P, Craig J. Consolidated criteria for reporting qualitative research (COREQ): a 32-item checklist for interviews and focus groups. *International Journal for Quality in Health Care*. 2007. Volume 19, Number 6 pp. 349 - 357
