# Supplementary material for: Barriers and Facilitators in the Implementation of the Systematic Medical Appraisal, Referral, and Treatment (SMART) Mental Health Digital Intervention in Rural India: Mixed Methods Process Evaluation Study
Source: JMIR Ment Health. 2026 May 7;13:e89164. doi: 10.2196/89164 (PMC13195372; doi:10.2196/89164)
Supplement: Multimedia Appendix 2 [file mental_v13i1e89164_app2.docx]

**Codebook for qualitative data analysis**

Title: Barriers and Facilitators in implementation of a digital mental health intervention in rural India: Findings from the Process Evaluation of the SMART Mental Health cRCT

| **Coding Category** | **Codes** |
| --- | --- |
| Context | About local context |
|  | People's perception of mental illness (before  intervention) |
|  | Perception/experience of stigma |
|  | Common stressors |
|  | Coping mechanisms |
|  | Adaptations to project |
|  | Reasons for adaptations/making changes |
| Barriers and Facilitators | Barriers in Implementation-Anti Stigma |
|  | Barriers in Implementation-M Health |
|  | Facilitators in Implementation-Anti Stigma |
|  | Fcilitators in Implementation-M Health |
| Perception of Intervention Effectiveness | Overall comments on SMH project |
| Perception of Intervention Effectiveness- (a)AntiStigma | Community/Patient Perception on utility.efectiveness-Anti Stigma-Positive |
|  | Examples demonstrating limited impact on knowledge, attitude, behaviour related to mental healht |
|  | Recall of anti stigma IEC |
|  | Perception of most effective IEC material |
|  | Community/Patient Perception on utility.efectiveness-Anti Stigma-Negative |
|  | ASHA's perception on utility/effictiveness-Anti Stigma-Positive |
|  | ASHA's perception on utility/effictiveness-Anti Stigma-Negative |
|  | Doctor's perception on utility/effectiveness-Anti Stigma-Positive |
|  | Doctor's perception on utility/effectiveness-Anti Stigma-Negative |
|  | Unsure about benefits of anti-stigma |
|  |  |
| (b)mHealth (Screening, camps, OPD, referral,  ASHA follow-up) | Community/Patient Perception on utility.efectiveness-mHealth-Positive |
|  | Community/Patient Perception on utility/effectiveness-mHealth-Negative |
|  | ASHA's perception on utility/effictiveness-mHealth-Positive |
|  | ASHA's perception on utility/effictiveness-mHealth-Negative |
|  | Doctor's perception on utility/effectiveness-mHealth-Positive |
|  | Doctor's perception on utility/effectiveness-mHealth-Negative |
|  | Unsure about benefits of mHealth |
|  | About ASHA screening |
|  | Health Camps |
|  | Project staff's perception of effectiveness |
|  | Reasons for patient refusal to get treatment (at PHC and camp) (intervention) |
|  | Strategies to address patient refusal |
|  | Perception about benefits of having participated in  the project |
| IVRS | Perception about IVRS |
| Trainings | Perception about trainings |
| Intervention Acceptability and Adoption (EDSS) | Perception of ASHAs about EDSS- Positive |
|  | Perception of ASHAs about Asha EDSS- Negative/Challenges |
|  | Perception of Doctors about EDSS-Positive |
|  | Perception of Doctors about EDSS-Negative/Challenges |
|  | Facilitators in use of EDSS (both ASHAs and Docs) |
|  | Time taken to examine a patient |
|  | Confidence in prescribing medicines |
| Health Service Use | Barriers in accessing care at PHC (int +control) |
|  | Experience with PHC doctor(intervention) |
|  | Experience with psychiatrist |
|  | Facilitator in accessing care at PHC |
|  | Perception of Teleconsultation |
| Future Directions | Barriers to scale up |
|  | Facilitators to scale up |
|  | Suggestions for improving implementation in future |
|  | Suggestions on improving anti stigma |
|  | Perception about existing government programmes |
